# Tuberculosis of the Lateral Condyle of the Femur: A Case Report

**DOI:** 10.7759/cureus.48658

**Published:** 2023-11-11

**Authors:** Rajavel K, Eswaran Mythilisri, Gowtham S, Saravanan V

**Affiliations:** 1 Department of Orthopaedics, Sri Ramaswamy Memorial (SRM) Medical College Hospital and Research Center, Chennai, IND

**Keywords:** single foci, adult, lytic lesion, lateral femoral condyle, skeletal tuberculosis

## Abstract

Tuberculosis in India has been a constant health issue. The revised national tuberculosis control program has suggested antitubercular drug regimens according to WHO guidelines for pulmonary and extrapulmonary tuberculosis. Here is an uncommon case of an adult presenting with a single lytic lesion of the lateral condyle of the femur; he had no history of primary foci of pulmonary tuberculosis and curettage of the lesion and histopathological examination of the bone tissue showed tubercular etiology. The patient, hence, was started on antitubercular drugs and at a six-month follow-up, resolution of the lesion was noticed.

## Introduction

Tuberculosis remains a major international health problem and the leading cause of avoidable death despite advances in radiological diagnosis and antituberculous therapy as people often do not seek care due to poor knowledge of the disease, stigma, or health access barriers [1,2,3]. Extrapulmonary tuberculosis cannot be ruled out with a negative tuberculin test or clear chest X-ray. Skeletal tuberculosis results from the implantation of *mycobacteria* in the medulla of the bone. It is most common in the spine followed by the femur, tibia, and small bones of hands and feet, requiring CT or MRI imaging and histopathological examination for diagnosis. Though the incidence of skeletal tuberculosis has decreased among the European population, it is still on the rise among Asian, African, and American populations. The metaphysis is the most common site of the initial lesion, which could either be contained or spread to the epiphysis, where it could affect the adjacent joint. Skeletal tuberculosis affecting the metaphysis in long bones is more commonly noted in children than in adults. Also disseminated tuberculous infection affecting a single bone and manifesting as focal lytic cortical lesions is rare and unusual [2,4]. The odd sites of skeletal tuberculosis are the sternum, ribs, sternoclavicular joint, and calvaria [5]. In this case report, we present a rare case of focal lytic lesion in the lateral condyle of the femur of an adult.

## Case presentation

**Figure 1:**
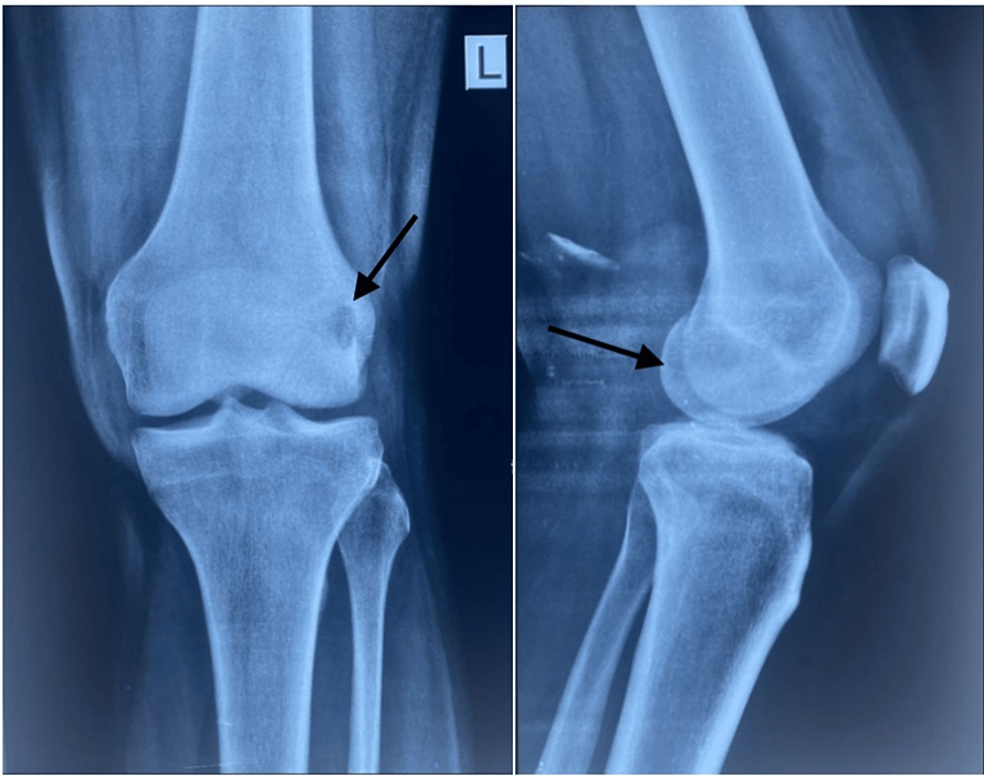
Preoperative X-ray

A 28-year-old male with no known comorbidities presented with complaints of pain in his left knee for one month associated with difficulty in walking for three weeks. He was apparently normal two months back when he had an alleged history of road traffic accident (two-wheeler vs two-wheeler) and sustained an injury to his left knee, following which he was asymptomatic and was able to bear weight. Five weeks following the incident, he developed pain in his left knee along with difficulty in weight bearing. He gave a history of fever going one month back, which settled with antipyretics. He did not have any history of constitutional symptoms such as loss of appetite, loss of weight, evening rise in temperature, cough, or cold. There was a family history of his grandmother having a similar lytic lesion in her left ankle thirty years back. On local examination of the left knee, a swelling of size 5x5cm was found present over the lateral condyle of the femur associated with tenderness; the active and passive range of movement of the knee was 0-60 degrees. Routine laboratory investigations were taken and were within normal limits. Erythrocyte sedimentation rate and C reactive protein values were elevated. Chest X-ray was normal. X-ray of the left knee showed a hypodense lesion in the lateral condyle of the femur (Figure 1).

**Figure 2:**
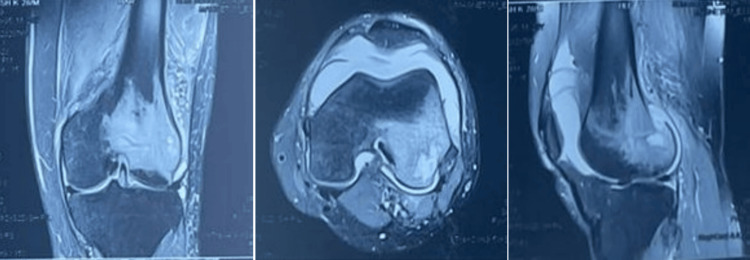
Preoperative MRI

MRI of the left knee further showed a hypoechoic area with erosion in the lateral femoral condyle along the epi-metaphyseal region with areas of cortical break posteriorly with permeative bone destruction, severe marrow edema involving the meta diaphyseal region of lower shaft of femur on lateral aspect extending to epiphysis surrounding the lytic lesion, all features favoring osteomyelitis with lytic lesion in lateral femoral condyle (Figure 2).

**Figure 3:**
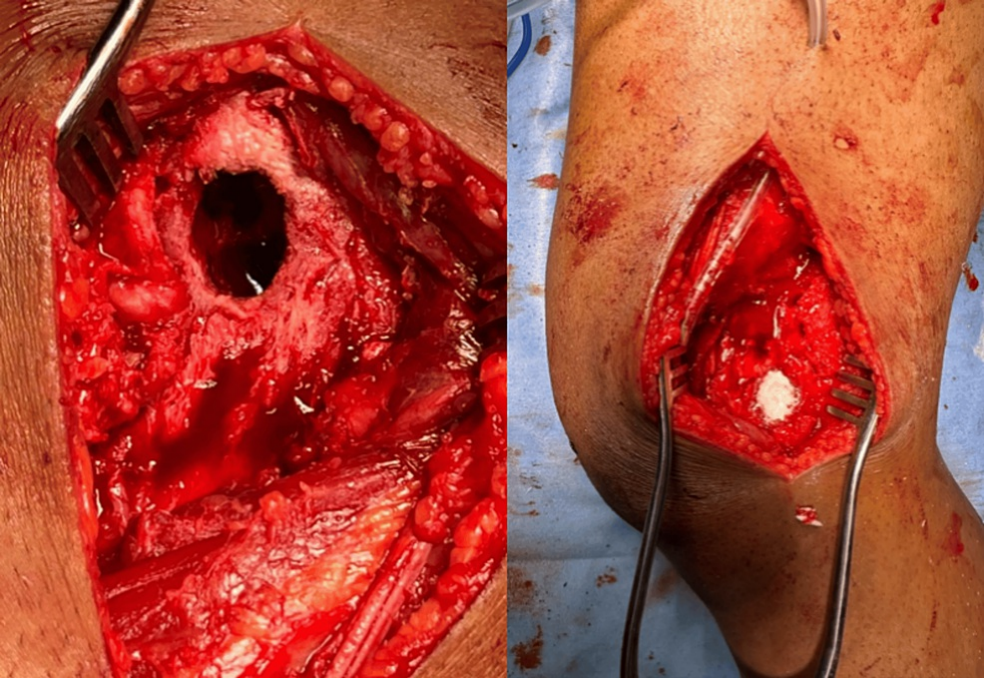
Intraoperative picture

The patient was taken up for excision biopsy and curettage where through lateral approach skin and soft tissue were dissected, the iliotibial band was also dissected and vastus lateralis was split. The bone site was reached and the lytic lesion was visualized under C-arm guidance. Multiple drill holes were made over the lytic lesion site and curettage was done. A thorough wash was given, antibiotic vancomycin was kept over the curetted lytic lesion region, and the wound was closed in layers (Figure 3).

**Figure 4:**
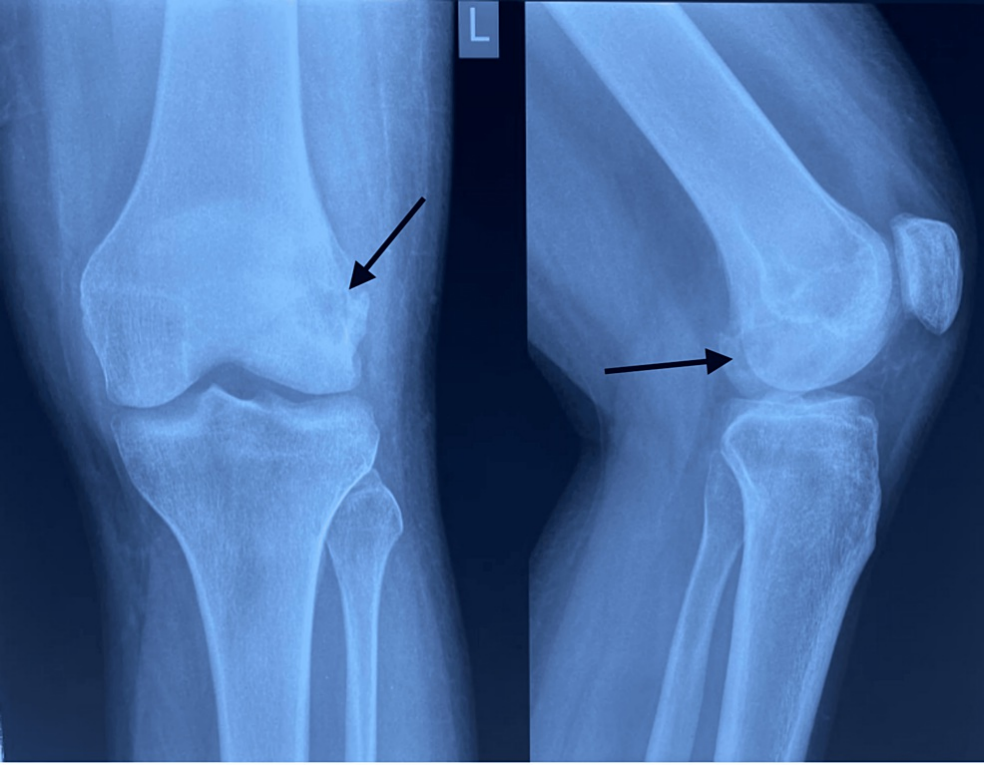
Postoperative X-ray

The curetted part of the bone was sent for acid-fast staining, gram staining, and culture sensitivity which turned up to be negative for any growth. Histopathological examination done on the curetted bone sample suggested features of necrotizing epithelioid granulomatous inflammation, showing the possibility of tuberculous etiology. He was hence started on category 1 antitubercular drugs under the Revised National Tuberculosis Control Programme as per revised WHO guidelines and followed up for six months. He was also started on physiotherapy such as static quadriceps exercise, knee bending exercise, and non-weight-bearing walking with walker support, which was gradually converted to partial weight-bearing walking after one week of post operative period. One month postoperative X-ray of the left knee showed a resolving lesion on the lateral condyle of the femur (Figure 4).

**Figure 5:**
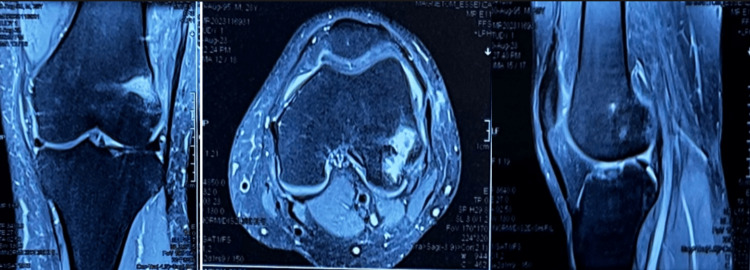
Postoperative MRI

MRI of the left knee was taken at the end of six months, which showed no new lesions (Figure 5).

**Figure 6:**
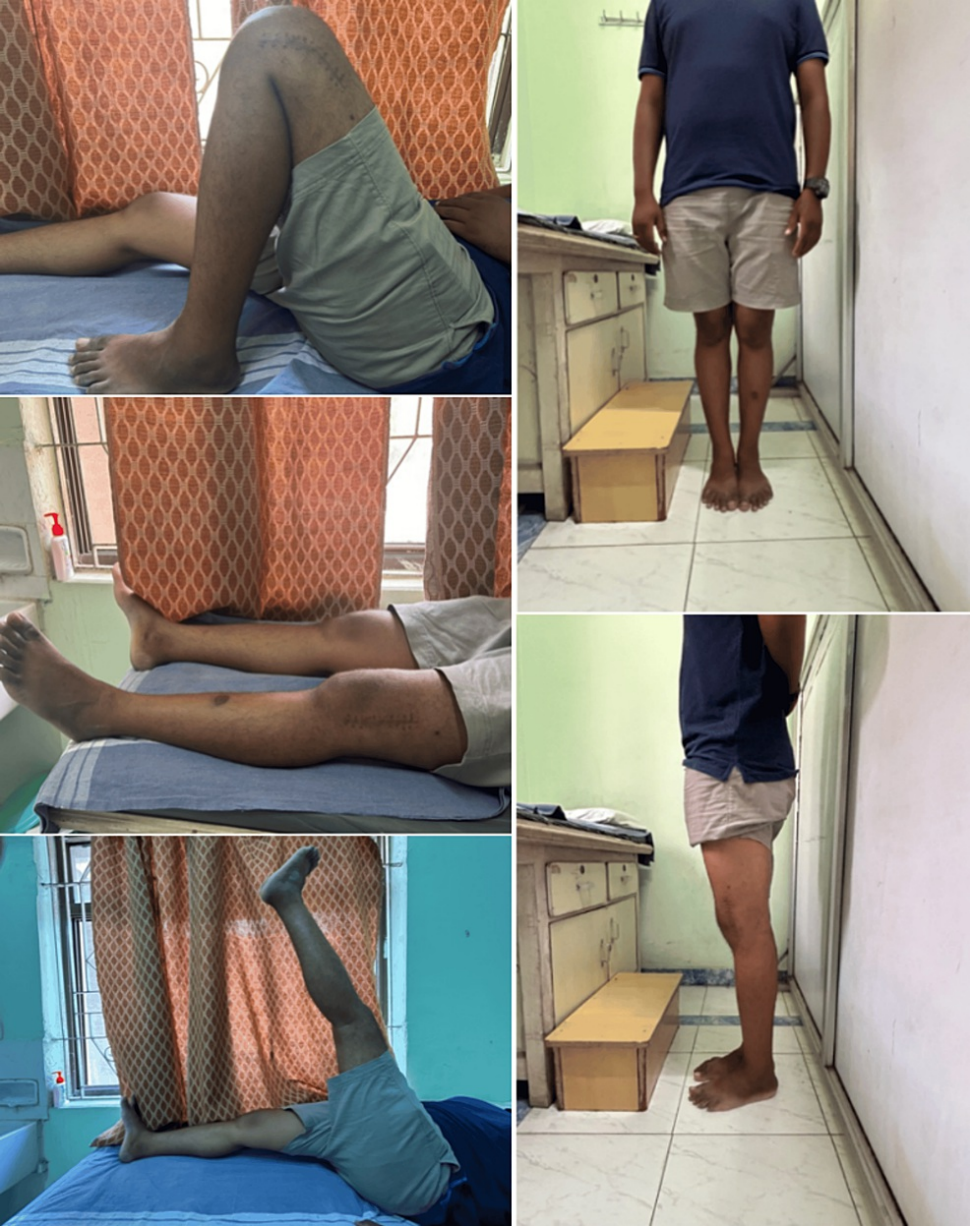
Postoperative range of movements of the left knee

He has improved symptomatically with the full range of movements of the left knee (Figure 6) and is able to walk with full weight bearing.

## Discussion

Tuberculosis infection has one of the highest mortality and morbidity rates in the world. An estimated 10.6 million people are infected with tuberculosis every year among which one in five present with extrapulmonary tuberculosis of which 6% amounts to skeletal tuberculosis [6,7]. Ten percent of all skeletal tuberculosis presents in the knee. It is the third most common site of osteoarticular tuberculosis. Early diagnosis of skeletal tuberculosis is essential to reduce the risk of deformity. MRI and CT have enhanced the diagnosis of musculoskeletal tuberculosis and have assisted biopsies from the affected site. Leonard et al. noticed that 30% of cases of musculoskeletal tuberculosis occurred during the second decade of life, 22% in the first decade, 18% in the third decade, and 14% in the fourth decade, and noticed that a closed cystic form of skeletal tuberculosis can occur in the long bones, which may not be associated with sclerosis or osteopenia or abscess/sinus tract formation as in other forms of skeletal tuberculosis. This form of tuberculosis is more likely to occur in children and may be misdiagnosed as a malignancy, but in our case report, we have found a similar lesion in a young adult [8]. 

Baykan et al. have described tuberculous osteomyelitis affecting the femur, tibia, and the bones of hands and feet, having primary foci in the lungs, which may or may not be an active disease, mainly spreading to the epiphyseal region in the pediatric age group [6], whereas, in our study, the lytic lesion was noticed in the epiphyseal and metaphyseal regions of an adult with no evidence of an active or healed pulmonary tuberculosis. Birole et al. and Kori et al. have noticed multiple lytic lesions in the long bones of the adult age group [9,10]; however, our case presented a single lytic lesion in the lateral condyle of the femur. Almost all the studies on skeletal tuberculosis have had patients presenting with constitutional symptoms, but here we present a case of an adult without any constitutional symptoms who was diagnosed with tubercular osteomyelitis only with the histopathological report and started on antitubercular drugs. Araujo et al. have presented a case with multifocal skeletal tubercular lesions which were treated with antitubercular drugs for 30 months until clinical improvement [4], whereas we treated our patient for six months with the category 1 antitubercular drugs, following which the patient showed good recovery with resolution of the lytic lesion in X-ray and clinical improvement by being able to continue daily activities similar to prediagnostic state.

## Conclusions

Tuberculosis is very common in India but its presentation as a single lytic lesion in the lateral femoral condyle in an adult in the absence of other bone and lung lesions is very unlikely. The likely chance of an orthopedist encountering and treating tuberculosis in atypical sites in long bones warrants the need for suspicion and early diagnosis as the risk of multidrug resistance is on the rise. Our case highlights the high degree of suspicion one must have in diagnosing a lytic lesion of long bones. Error in diagnosis and treatment burdens the medical resources and overall morbidity. Hence, tuberculosis must be kept in mind as one of the differential diagnoses for a lytic lesion in the skeletal system until proven otherwise even if the patient might not present with any constitutional symptoms.
